# Case report: Subacute transverse myelitis with gait preservation secondary to Lyme disease and a review of the literature

**DOI:** 10.3389/fped.2023.1064234

**Published:** 2023-03-16

**Authors:** Charlotte Colot, Catherine Adler, Céline Mignon, Alessandro De Leucio, Patrice Jissendi, Jean Fonteyne, Alec Aeby

**Affiliations:** ^1^ Service de Neurologie Pédiatrique, Hôpital Universitaire des Enfants Reine Fabiola (HUDERF) et Hôpital Erasme—Hôpital Universitaire de Bruxelles (HUB)—Université Libre de Bruxelles (ULB), Bruxelles, Belgium; ^2^ Clinique d’infectiologie, Centre Hospitalier Universitaire (CHU) Saint Pierre, Bruxelles, Belgium; ^3^ Clinique d’infectiologie, Hôpital Universitaire des Enfants Reine Fabiola (HUDERF) et Hôpital Erasme—Hôpital Universitaire de Bruxelles (HUB)—Université Libre de Bruxelles (ULB), Bruxelles, Belgium; ^4^ Service de Radiologie, Hôpital Universitaire des Enfants Reine Fabiola (HUDERF) et Hôpital Erasme—Hôpital Universitaire de Bruxelles (HUB)—Université Libre de Bruxelles (ULB), Bruxelles, Belgium; ^5^ Service de Radiologie, Centre Hospitalier Universitaire (CHU) Saint Pierre, Bruxelles, Belgium; ^6^ Service de Pédiatrie, Hôpital Universitaire des Enfants Reine Fabiola (HUDERF) et Hôpital Erasme—Hôpital Universitaire de Bruxelles (HUB)—Université Libre de Bruxelles (ULB), Bruxelles, Belgique

**Keywords:** transverse myelitis (TM), Lyme disease (LD), subacute, gait preservation, review

## Abstract

Subacute presentation with gait preservation is rare in the initial presentation of transverse myelitis (TM) in children. Lyme TM is poorly described in the literature. Here, we present the case of a 10-year-old boy who presented with neck pain with irradiation in the upper limbs for 13 days, accompanied by a right latero-torticollis. Magnetic resonance imaging (MRI) of the spine showed a hypersignal in the centromedullary T2 weighted image (WI) between C1 and C7, which was suggestive of cervical TM. A lumbar puncture revealed pleocytosis and proteinorachia. The test results of *Borrelia* IgG in the blood and intrathecal IgG synthesis were positive, confirming the diagnosis of TM secondary to Lyme disease. The patient was treated with high doses of steroids and antibiotics, following which he recovered completely. After a review of the clinical features of the eight previously published pediatric cases, we can conclude that Lyme TM usually has a subacute clinical presentation and is frequently limited to the cervical spine with pure sensory symptoms and gait preservation. Moreover, acute and chronic sphincter dysfunction is rare, and recovery is usually complete.

## Introduction

Transverse myelitis (TM) is a rare disorder with approximately 1–5 new cases per million of the population per year, with 20% of all cases reported in patients under 18 years of age (1). Diagnosis requires clinical symptoms and evidence of inflammation within the spinal cord *via* cerebrospinal fluid analysis and/or magnetic resonance imaging. The onset of symptoms varies from 4 h to 3 weeks, but in almost half of the cases, the diagnosis is made within 24 h (1). Clinical features consist of sensory disturbances in most patients, followed by weakness and sphincter dysfunction. Children suffer from more severe clinical impairment than adults. In the series by Pidcock et al., 89% of the pediatric patients were bed- or wheelchair-bound or required assisted ventilation (2). Treatment methods include oral steroids, intravenous immunoglobulins, plasma exchange, and immunomodulatory therapies. Sensory-motor symptoms and chronic sphincter dysfunction have been shown to be the principal sequelae.

Lyme disease is an infection secondary to a bacterium of the *Borrelia*-type spirochete family transmitted by Ixodes ticks. The most common species in Europe are *Borrelia afzelli* and *garinii*, which are responsible for more neurological damage. Natural evolution occurs in three stages. The primary attack results in the appearance of an erythema migrans from a few days to a few weeks after the bite, accompanied by non-specific general signs. The secondary phase is revealed by an attack that can be multisystemic: visceral, ophthalmological, cardiological, rheumatological, and neurological (which can be central or peripheral). The most common condition in children is peripheral facial paralysis. The last phase is manifested by more severe neurological damage such as myelopathy, progressive encephalopathy, or ischemic stroke. Neurological manifestations occur in 3%–15% of all cases of Lyme disease (3). The treatment requires antibiotic therapy and the prognosis is usually excellent, particularly in children. The diagnostic evaluation for neuroborreliosis should always include a cerebrospinal fluid (CSF) lumbar puncture, leukocyte cell count, and measurement of the *B. burgdorferi* CSF-to serum antibody index. However, a negative antibody index has been found in up to 30% of neuroborreliosis patients. In these patients, the typical clinical symptoms, the presence of *B. burgdorferi* IgM and IgG antibodies in the serum, CSF pleocytosis, and a positive response to antibiotic treatment are mandatory criteria for the diagnosis of neuroborreliosis (4).

TM secondary to Lyme disease accounts for 4% of all neurological damage secondary to Lyme disease (4). Eight pediatric cases have been reported so far. We present the ninth pediatric case, and after an extensive review of the literature, we could provide evidence that the clinical presentation of neuroborrelial TM is different from classical TM. Indeed, TM secondary to Lyme disease is more often subacute with gait preservation, is limited to the cervical spine, sphincter dysfunction is unusual, and recovery is complete after prolonged antibiotherapy. These findings may help improve the management of TM in children.

## Case report

A 10-year-old boy with no particular medical history attended a consultation with his pediatrician because of persistent nocturnal and rotational neck pain with irradiation in the upper limbs for 13 days with a feeling of heaviness and paresthesia in the fingers. Pyrexia was also present for 11 days (38–39°C) with fatigue and headaches. A clinical examination revealed pain provoked by anteroposterior and lateral flexion of the neck accompanied by a right latero-torticollis and dysesthesia in the C3–C4 level on the right side. The cranial nerves, cerebellar tests, motor strength, and walking gait were normal, and the patient had no sphincter problems. Vital parameters were normal, as were the results of a physical examination.

**Figure 1:**
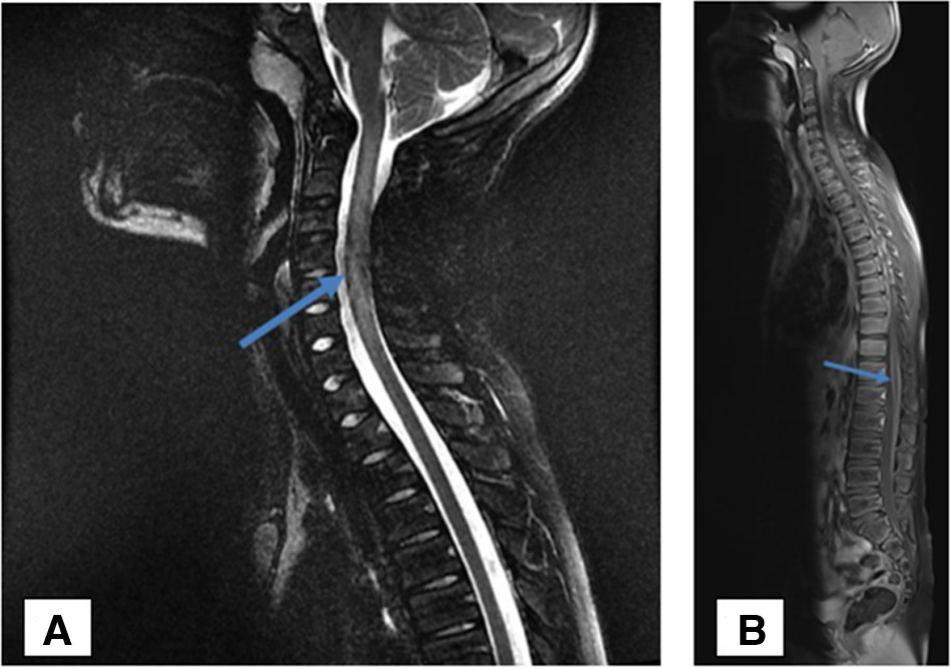
MRI of the cervical spine showing extensive centromedullary T2 weighted image (WI) hypersignal (**A**) with normal T1 WI signal impairment between the C1–C7 metamers associated with mild swelling at the cervical spinal cord and leptomeningeal enhancement at the lumbosacral level and the cauda equine (**B**).

Magnetic resonance imaging (MRI) of the cervical spine revealed a C1–C7 centromedullary T2 weighted image (WI) hypersignal with a normal T1 WI signal associated with a slight swelling of the cervical cord and a leptomeningeal enhancement at the lumbosacral and the cauda equina level (Figure 1) on T1 WI with gadolinium injection, suggestive of longitudinal extensive transverse myelitis (LETM).

Therefore, an extensive workup was performed using blood serologies (*Mycoplasma*, *Treponema*, *Hepatitis*, *Borrelia*, *Herpes*, *Human immunodeficiency virus*, and *West Nile)*, autoimmune factors (antinuclear factor, anti-neutrophil cytoplasm antibodies, anti-ganglioside and rheumatoid factor), Myelin oligodendrocyte glycoprotein (MOG), anti-aquaporin 4 (AQP4), onco-neuronal antibodies, CSF analysis with HSV1 polymerase chain reaction, viral meningitis/encephalitis panel (*Haemophilus influenzae, Listeria monocytogenes, Neisseria meningitidis, Streptococcus agalactiae, Streptococcus pneumoniae, Cytomegalovirus, Enterovirus, Herpes simplex virus 1, Herpes simplex virus 2, Human herpes virus 6, Parechovirus,* and *Varicella zoster virus et Cryptococcus neoformans/gattii*) and oligoclonal bands.

**Figure 2:**
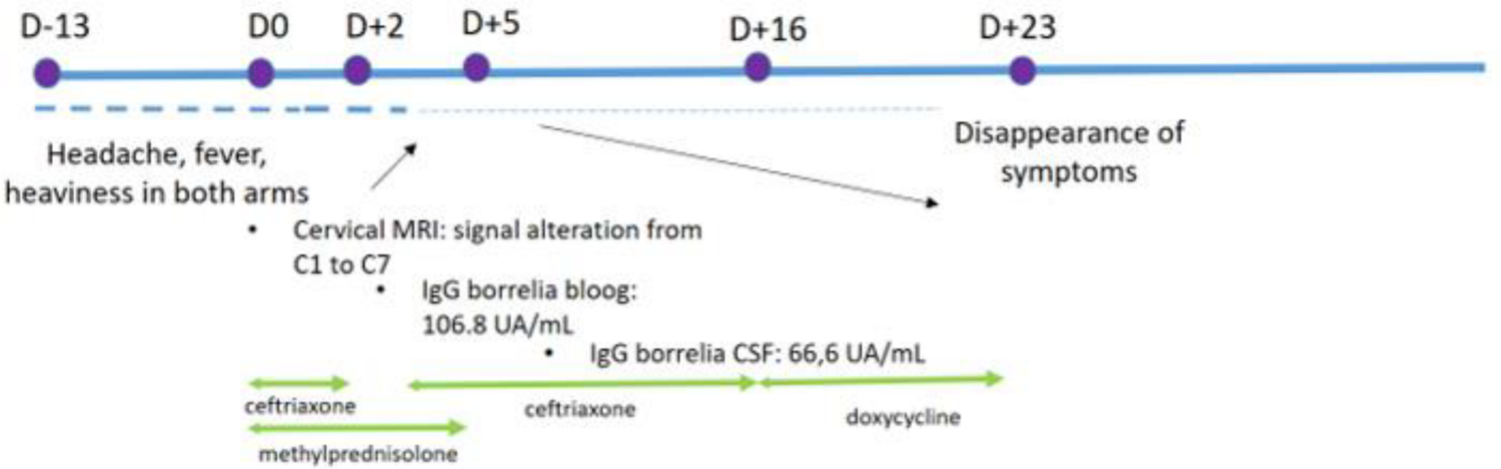
Summary of the clinical case in the presentation of a timeline. D0 corresponds to the diagnosis of transverse myelitis; ↗ to an increase in symptoms; ↘ to a decrease in symptoms.

A CSF analysis revealed a pleocytosis (268 nucleated elements/µL; normal value: 0–5/µL) with 5.6/µL red blood cells (normal value: 0–5/µL) and proteinorachia (2.36 g/L; normal value: 0.15–0.45 g/L), and the meningitis/encephalitis panel was negative. The patient was treated with high-dose methylprednisolone 1.2 g/day (=30 mg/kg/day) IV for 5 days and Ceftriaxone 2 g 2×/day IV (=100 mg/kg/day). After 48 h, the symptoms decreased and CSF bacterial culture was negative, and, therefore, Ceftriaxone was stopped. Nevertheless, after 2 days under steroids alone, neck pain and laterocollis reappeared. Because the patient was a resident of a tick-endemic area, blood *Borrelia* IgG was tested by using ELISA, and the results were positive (106.8 N: 10–15 UA/mL), which was confirmed by Western blot. The patient reported that he had an erythematous spot in the neck a few months back, which was suggestive of an erythema migrans, but he did not remember that he had suffered from a tick bite. Ceftriaxone was restarted, and the day after, a CSF *Borrelia* IgG test was done, which showed positive results (IgG: 66.6 UA/mL—normal value: 4.5–5.5 UA/mL), with oligoclonal bands in the CSF confirming the diagnosis of neuroborrelial TM. The patient received Ceftriaxone 100 mg/kg/day IV for a total of 14 days, followed by oral Doxycycline 200 mg 1×/day for 7 days. On day 7 of his hospitalization, a spinal MRI was performed, which showed a normalization of cervical spinal cord signal abnormalities and a complete regression of leptomeningeal enhancement in the cauda equina. The patient recovered completely after 23 days (Figure 2).

## Discussion

TM is a rare and potentially devastating medical condition with variable outcomes. TM secondary to Lyme disease are exceptionnal and limited to the description of single cases, both in children and adults. After an extensive review of the pediatric literature, we wish to emphasize five aspects of TM secondary to Lyme disease: (1) presentation is more often subacute, (2) lesions are mainly located in the cervical spine, (3) gait is usually preserved, (4) sphincter dysfunction is unusual, and (5) recovery is usually complete after prolonged antibiotherapy.

Our patient developed a subacute TM with pain provoked by anteroposterior and lateral flexion of the neck, accompanied by a right latero-torticollis and dysesthesia at the C3–C4 level on the right side. A spinal MRI showed an extensive T2 hypersignal between C1 and C7, with a normal T1 signal confirming the diagnosis of LETM. Even if the patient did not spontaneously complain of a tick bite, we tested *Borrelia* IgG by ELISA as he was a resident of a tick-endemic area. The *Borrelia* IgG test results were positive in the blood (106.8 UA/mL—normal value: 10–15 UA/mL), which was confirmed by Western Blot, and CSF *Borrelia* IgG test results were also positive (IgG: 66.6 UA/mL—normal value: 4.5–5.5 UA/mL), confirming the diagnosis of TM secondary to Lyme disease. Various hypotheses were excluded for our patient. Because encephalopathy was not a part of the initial clinical presentation, a diagnosis of acute disseminated encephalomyelitis (ADEM) was unlikely. Multiple sclerosis (MS) was not evoked because the brain MRI did not meet McDonald's criteria, and the spinal MRI did not show small segment plaques within the cervical cord but did show LETM. Neuromyelitis Optica was excluded because the patient had no optic involvement, and the test results of AQP-4 and MOG antibodies in the blood were negative. The diagnosis of flaccid myelitis was not evoked because the patient had no flaccid paresis and there were no signal abnormalities in the gray matter on the spinal MRI. The patient does not fall within the framework of a Myelin oligodendrocyte glycoprotein antibody disease (MOGAD) in a broader sense because the results of the anti-MOG antibody test were negative. The results of the tests of the antinuclear factors and ANCA were also negative, which did not suggest an autoimmune disease. Neither the clinical presentation nor the negative onconeuronal antibodies were suggestive of a paraneoplastic disease.

We found eight pediatric cases of neuroborrelial TM described in the literature in addition to our case (Table 1) (4–6) and compared them with the Pidcock review that included five pediatric series (2) for a total of 125 patients with TM. First, we could observe a longer delay in symptoms for TM secondary to Lyme disease (17 days + 11.5SD) compared with 2 days for the other causes of TM in children. Subacute presentation has already been reported in neuroborreliosis with different presentations such as cerebellitis (7), meningitis, and radiculoneuritis. Even though acute presentations of TM have also been described [68.2% of cases were assigned a diagnosis of TM within 7 days of the onset of symptoms (2)], a subacute neurological presentation might be a clue in the diagnosis of neuroborrelial TM. The *B. burgdorferi* genome does not encode any known toxins or the machinery that would be required to secrete them; tissue damage, and hence disease, is believed to be mediated by the inflammatory response elicited in the mammalian host (3). This may be the reason why the involvement is subacute in Lyme disease. Little is understood about the processes that occur late in infection and the mechanisms that enable the bacterium to persist in the face of the robust cellular and humoral immune responses that it elicits. In the past, the emphasis has been on immune evasion by the spirochaete, and this topic needs greater scrutiny. Nevertheless, attention also needs to be paid to the possibility that host-mediated modulation of pathogen-sensing pathways and of resultant responses contributes to spirochaete persistence (8). Second, in neuroborrelial TM, involvement of the spine is mostly cervical in six out of nine patients (66%) while in the Pidcock review, cervical involvement was present in only 14% of patients (2). Interestingly, as in the case of Bigi et al., there was also a striking discrepancy between the severe mainly cervical imaging manifestations and the minimal clinical signs and symptoms (9). As the presence of a- or pauci-symptomatic TM is known to predict multiple sclerosis and neuromyelitis optica (NMO) (1), our case report might also suggest that neuroborrelial TM should be suspected in paucisymptomatic TM. Third, the inability to walk is seen in more than 80% of TM in children (2), while it is observed only in two out of nine patients (22%) in neuroborrelial TM. Gait preservation in neuroborrelial TM might be explained by the predominance of the cervical spine lesion but also by the discrepancy between the clinical manifestations and the extent of the spinal MRI abnormalities. Fourth, sphincter dysfunction is present in 87% of patients in the Pidcock series, while it is documented in just one out of nine (11%) patients in TM secondary to Lyme disease (2). In pediatric TM, a high dose of corticosteroids is the recommended treatment for TM in children and adults. Nevertheless, our patient first showed improvement on combined steroids and antibiotherapy, but his condition deteriorated on steroids alone after we stopped antibiotherapy because of a negative CSF bacterial culture, and finally, he was cured with long-term antibiotherapy. Our case illustrates that neuroborrelial TM should be treated with long-term antibiotherapy and that steroids do not seem to improve the prognosis.

**Table 1:** Summary table of the different cases of patients with transverse myelitis caused by Lyme disease described in the literature, to which our patient case has been added. In this study, we were interested in determining the following: duration of symptoms, localization, gait preservation, sphincter disorder, response to treatment.

Reported cases	Duration of symptoms (days)	Localization	Gait preservation	Sphincter dysfunction	Response	Treatment
Baumann et al. (10)	21	From the pons down to C5	Yes	No	Complete recovery	Céfalosporine and NSAIDs
Meurs et al. (11)	14	Cervical spinal cord from C3 to C7	Yes	No	Complete recovery	Ceftriaxone (1.5 g/day during 3 weeks) and methylprednisolone (5 days 750 mg/day)
Huisman et al. (12)	5	Cervical spinal cord	Yes	No	Responsive (information about full or partial response could not be obtained from the report)	Ceftriaxone IV
Linssen et al. (13)	3	—	Difficulty in walking	Yes	Complete recovery	Penicilline IV
Bigi et al. (9)	28	Medulla down to cervical spine—pons to the conus	Yes	No	Complete recovery	Ceftriaxone IV (14 days)
Erol et al. (4)	10	T10-L1	Weakness in both legs	No	Complete recovery	Methylprednisolone IV (1 g/day for 5 days) followed by prednisolone for 1 month and 4 mg/kg of doxycycline for 14 days
Khan et al. (5)	21	T7 to the conus	Yes	No	No information	Ceftriaxone 2 g 2×/day for 21 days
Gaudichon et al. (6)	42	C2 to C5	Yes	No	Complete recovery	Ceftriaxone 2 g 2×/day for 28 days
Our Case	13	C1 to C7	Yes	No	Complete recovery	Methylprednisolone IV 5 days and Ceftriaxone 2 g/day IV for 19 days and doxycycline for 7 days

The limitation of our article lies in the fact that we extrapolate information from a limited group of patients (nine cases) because TM secondary to Lyme disease is rare and not all cases are reported in the literature.

## Conclusion

TM in a subacute presentation, gait preservation, a discrepancy between the severe mainly cervical imaging manifestations and the minimal clinical signs and symptoms, and the absence of sphincter dysfunction should raise suspicion of TM secondary to Lyme disease. Those with TM presenting with the above characteristics should be maintained on antibiotherapy until the results of Lyme serology are obtained even in the presence of a negative CSF culture. Prognosis is usually excellent after a 3-week antibiotherapy, with usually complete recovery.

## Data Availability

The original contributions presented in the study are included in the article/Supplementary Material, and further inquiries can be directed to the corresponding author.
